# Neurite orientation dispersion and density imaging reveals abnormal white matter and glymphatic function in active young boxers

**DOI:** 10.1002/ejsc.12113

**Published:** 2024-04-26

**Authors:** Wenjing Huang, Laiyang Ma, Jiahao Yan, Wanjun Hu, Guangyao Liu, Rui Wang, Jing Zhang

**Affiliations:** ^1^ Department of Magnetic Resonance Lanzhou University Second Hospital Lanzhou China; ^2^ Second Clinical School Lanzhou University Lanzhou China

**Keywords:** injury and prevention, neuroscience, quantitative study

## Abstract

The neurological effects and underlying pathophysiological mechanisms of sports‐related concussion (SRC) in active young boxers remain poorly understood. This study aims to investigate the impairment of white matter microstructure and assess changes in glymphatic function following SRC by utilizing neurite orientation dispersion and density imaging (NODDI) on young boxers who have sustained SRC. A total of 60 young participants were recruited, including 30 boxers diagnosed with SRC and 30 healthy individuals engaging in regular exercise. The assessment of whole‐brain white matter damage was conducted using diffusion metrics, while the evaluation of glymphatic function was performed through diffusion tensor imaging (DTI) analysis along the perivascular space (DTI‐ALPS) index. A two‐sample *t*‐test was utilized to examine group differences in DTI and NODDI metrics. Spearman correlation and generalized linear mixed models were employed to investigate the relationship between clinical assessments of SRC and NODDI measurements. Significant alterations were observed in DTI and NODDI metrics among young boxers with SRC. Additionally, the DTI‐ALPS index in the SRC group exhibited a significantly higher value than that of the control group (left side: 1.58 vs. 1.48, *P*_FDR_ = 0.009; right side: 1.61 vs. 1.51, *P*_FDR_ = 0.02). Moreover, it was observed that the DTI‐ALPS index correlated with poorer cognitive test results among boxers in this study population. Repetitive SRC in active young boxers is associated with diffuse white matter injury and glymphatic dysfunction, highlighting the detrimental impact on brain health. These findings highlight the importance of long‐term monitoring of the neurological health of boxers.

## Highlights

- Boxing is a contact sport involving direct physical impact, leading to the occurrence of repeated concussions and sub‐concussions among boxers throughout their careers; however, the routine detection of brain tissue lesions through CT and MRI remains a challenging task.
- Advanced magnetic resonance diffusion imaging diffusion MRI is performed on young active boxers to assess the extent of white matter damage and glymphatic function.
- The brains of active young boxers have extensive white matter microstructure damage and glymphatic dysfunction, which is associated with poorer cognitive performance.

## 1 INTRODUCTION

Sports‐related concussion (SRC) is a prevalent form of mild traumatic brain injury (mTBI) resulting from the biomechanics of collisions in contact sports, affecting up to 3.8 million individuals annually in the United States (Langlois et al., 2006). Boxing has been identified as one of the etiologies of SRC among athletes participating in high‐risk sports who may experience recurrent concussions and sub‐concussions. The acute phase of sub‐concussive impacts may not exhibit any discernible symptoms. However, these events have a cumulative impact that defies quantification and are believed to contribute to the development of chronic traumatic encephalopathy (Gallo et al., 2020; Pearce et al., 2020). Consequently, SRC frequently results in severe sequelae such as somatic symptoms (e.g., headache, dizziness, vestibular and oculomotor dysfunction, and fatigue) (Harris et al., 2022; Quinones et al., 2023), cognitive deficits (Teng et al., 2022), and psychomotor impairment (Ellis et al., 2015). Although most cases resolve within a few weeks of injury onset, some athletes may experience persistent symptoms lasting for months or even years. The neurological effects and underlying pathophysiological mechanisms of recurrent concussions and sub‐concussions remain poorly understood. Additionally, the 10–19 age group constitutes the largest cohort of athletes (Bryan, Rowhani‐Rahbar, Comstock, Rivara, & Seattle Sports Concussion Research, 2016), with approximately 70% of SRC occurring in young athletes in the United States (Coronado et al., 2015). The adolescent stage is a crucial period for the maturation of white matter in the brain. Brain damage during this stage can disrupt neurodevelopmental processes and result in cognitive impairment (Emery et al., 2016). However, the current absence of clinical biomarkers for objective diagnosis and prognosis determination of hinders evidence‐based treatment of concussed athletes.

Conventional MRI has limited sensitivity to brain microstructure, whereas diffusion magnetic resonance imaging (dMRI) offers unique insights into tissue microstructure. The principle is that dMRI can sensitively detect the diffusion of water molecules, and when the brain's white matter is damaged by concussion, the pattern of water molecule diffusion in the white matter is also affected. The past 2 decades have witnessed the emergence of diffusion tensor imaging (DTI) as a potent tool for characterizing white matter integrity in patients with mTBI and concussed athletes (Hellewell et al., 2021; Wu et al., 2016). However, DTI metrics derived from single‐compartment Gaussian models lack sensitivity to the complexity of white matter pathology and fail to directly reflect the biophysical properties of microstructures. In recent years, with the emergence of advanced dMRI models, neurite orientation dispersion and density imaging (NODDI) have demonstrated significant potential in characterizing cross fibers and providing a specific quantitative assessment of white matter pathology. NODDI is a non‐Gaussian model consisting of three compartments that quantify the diffusion behavior of water molecules within intracellular, extracellular, and free‐water environments. Three metrics of NODDI consist of the neurite density index (NDI), the orientation dispersion index (ODI), and the isotropic volume fraction (ISOVF). NDI represents the density of white matter axons, with a lower value indicating a decrease in axon density. ODI reflects the extent of neuronal dispersion, where 0 signifies perfectly straight‐aligned fibers and 1 indicates complete isotropy. ODI values are higher in loose tissues and lower in essentially parallel fibers such as those found in the corpus callosum. The ISOVF parameter indicates the proportion of free water in the tissue, and it has been suggested that the increase in ISOVF during the acute phase of concussion is indicative of vasogenic edema (Palacios et al., 2020). By using multiple b‐values, both DTI metrics and NODDI metrics can be calculated simultaneously (Zhang et al., 2011). A study conducted on concussed athletes demonstrated a decrease in NDI and an increase in ODI from the early symptomatic stages of concussion until the return to play (Churchill et al., 2019). Another study found that mixed martial artists with repeated concussions had decreased fractional anisotropy (FA) and increased NDI and ODI (Mayer et al., 2017). These inconsistent results have been attributed to variations in axonal physiology caused by the timing and the mechanism of injury. Furthermore, previous studies of SRC have focused on ball sports, whereas boxing, as a direct contact sport, has received relatively less attention due to its niche nature. Therefore, there is a need for further investigation into the neurological damage associated with boxing.

In addition to the white matter damage caused by repeated concussions, there is a potential risk of chronic damage to the glymphatic pathway. Recently, DTI analysis along the perivascular space (DTI‐ALPS) has demonstrated promising potential as a noninvasive tool for assessing glymphatic system function (Taoka et al., 2017). The glymphatic system refers to the space between astrocytic end feet and vessel walls, serving as an essential brain defense mechanism responsible for eliminating metabolic waste from the body (Iliff et al., 2012). Previous studies have utilized dynamic‐enhanced MRI with lumbar puncture intrathecal injection or intravenous gadolinium contrast agent to invasively investigate glymphatic function (Li et al., 2020; Ringstad et al., 2017). One study compared the outcomes of the ALPS index with classical dynamic‐enhanced MRI and discovered a significant correlation, further validating the feasibility of the DTI‐ALPS method (Zhang et al., 2021). The DTI‐ALPS approach is currently widely utilized in the assessment of Alzheimer's disease (Hsu et al., 2022), Parkinson's disease (Shen et al., 2022), and idiopathic normal pressure hydrocephalus (Georgiopoulos et al., 2023), all of which have consistently reported a decreased ALPS index. A study conducted on adult individuals revealed that the ALPS index exhibited a significantly lower value in patients with traumatic brain injury than healthy controls (HCs) (Jung Hyun Park et al., 2023). However, considering the ongoing development of the glymphatic system in young athletes, it remains unclear how recurrent sports‐related concussions (SRC) impact the functionality of their glymphatic system.

Therefore, the present study prospectively enrolled a cohort of active young boxers to investigate the impact of repetitive concussions and sub‐concussions on white matter integrity and glymphatic system functionality, with the anticipation of identifying potential imaging biomarkers. Tract‐based spatial statistics (TBSS) analysis was employed to compare the effectiveness of DTI and NODDI metrics in detecting abnormalities in white matter following SRC. We utilized region of interest (ROI)‐based analysis to evaluate the vulnerability of white matter fiber tracts following SRC. Subsequently, we compared the DTI‐ALPS index between boxers and controls to assess its potential as an indicator of glymphatic function. Finally, we examined the association between these metrics and clinical characteristics and neuropsychological performance and compared their sensitivity in detecting cognitive impairments.

## 2 METHODS

### 2.1 Study participants

Our scan protocol was approved by the Institutional Review Board, and written informed consent was obtained from each subject before scanning. The study recruited amateur boxers diagnosed with SRC from local sports teams, while healthy controls (HCs) with hobbies such as basketball, badminton, and long‐distance running were recruited through advertising. The control group was matched to the SRC group in terms of age and education. The inclusion criteria, as per the Consensus Statement on Concussion in Sport (McCrory et al., 2017) agreed upon by the SRC group meeting, are as follows: (i) the athlete has a history of direct or indirect head impacts; (ii) transient neurological impairment following a head impact, including confusion or disorientation, loss of consciousness or post‐traumatic memory loss, or focal symptoms such as seizures; and (iii) absence of significant trauma observed on conventional MRI. The exclusion criteria included: (i) a history of substance abuse; (ii) a history of neuropsychiatric disorders; and (iii) contraindications for MRI. In the case of HCs, exclusion criteria also included a history of head trauma. Boxers were interviewed to gather information on their specific sport types, duration in sports, number of previous concussions, and time elapsed since the last SRC. Three boxers were excluded from the study due to not meeting the inclusion criteria, and two boxers and two controls were excluded due to suboptimal imaging quality (Please refer to Supplementary Figure S1 for the flow diagram of the inclusion process). The final cohort consisted of 60 participants: 30 individuals with SRC who were boxers (sex: 11 females/19 males; age: mean = 20.57 years, SD ± 2.9; education: mean = 10.7 years, and SD ± 2.53), and a control group of 30 healthy individuals matched for age and education without any previous history of concussion (sex: 18 females/12 males; age: mean = 21.93 years, SD ± 4.23; education: mean = 11.7 years, and SD ± 3.22).

### 2.2 Neuropsychological assessment

The subjects underwent neuropsychological tests and other validated outcome measures, as documented in the existing literature, to evaluate common cognitive, emotional, and physical symptoms following SRC (Kinnunen et al., 2011). The following tests were assessed: Trail Making Test A and B (attention and cognitive flexibility), Trail Making Test B minus A (B−A, executive function), forward and backward digit span test (attention and working memory), the Rey Auditory Verbal Learning Test (RAVLT, scores on the sum of item 1‐5 of the word list evaluates language learning ability, scores on item 6 of the word list assess instantaneous memory, and scores on item 7 of the word list are measured 30 min later to assess delayed memory function), the Montreal Cognitive Assessment (MoCA, eight cognitive domains), the Hamilton Depression Scale (HAMD), Hamilton Anxiety Scale (HAMA) (affective symptoms), and the Post‐Concussion Scale (PCS, evaluates a range of commonly experienced symptoms following head trauma, encompassing cognitive, emotional, and physical domains).

### 2.3 MRI scanning

MRI data were acquired using a 3T MR scanner (SIGNA^TM^ Premier; GE Healthcare) equipped with a 48‐channel head coil. The NODDI data was obtained through a multi‐slice single‐shot spin‐echo echo‐planar image sequence (SE‐EPI) with TR = 5705 ms, TE = 68.8 ms, FOV = 240 mm, matrix = 120 × 120, 78 slices, and slice thickness = 2 mm. The NODDI protocol consisted of acquiring diffusion‐weighted images along three b‐values: twenty directions at *b* = 1000 s/mm^2^, forty directions at *b* = 1800 s/mm^2^, and sixty directions at *b* = 2500 s/mm^2^. The protocol also included a *b* = 0 s/mm^2^ image with a reversed phase‐encoding direction. The acquisition time for NODDI was 11 min and 42 s. Each participant underwent a Magnetization Prepared‐Rapid Gradient Echo imaging sequence (MP‐RAGE) to obtain standard high‐resolution sagittal three‐dimensional (3D) T1‐weighted images (T1WI). The imaging parameters were TR = 2632 ms, TE = 3000 ms, TI = 1000 ms, acquisition matrix = 256, 8° flip, 392 slices, and 1‐mm isotropic resolution over a scanning time of 6 min. These images were utilized for identifying abnormal brain structures.

### 2.4 MRI image analysis

#### 2.4.1 NODDI processing

The preprocessing and analysis of NODDI were conducted using the tools provided in the FMRIB Software Library (FSL, version 5.0.7, https://www.fmrib.ox.ac.uk/fsl). The acquired images were corrected for eddy current distortion and rigid‐body head motion, and skull‐stripped was performed based on the *b* = 0 s/mm^2^ image using the Brain Extraction Tool (Smith, 2002). Tensor fitting and DTI metrics (FA, mean diffusivity (MD)) were computed utilizing the FSL Diffusion Toolbox. Subsequently, axial diffusivity (AD) and radial diffusivity (RD) parameter images were generated for each participant. NODDI fitting was performed using the CUDA Diffusion Modelling Toolbox (cuDIMOT, https://users.fmrib.ox.ac.uk/~moisesf/cudimot/) running on Graphics Processing Units. Subsequently, images depicting NDI, ODI, and ISOVF were generated.

#### 2.4.2 TBSS analysis

The DTI and NODDI metrics, including FA, MD, AD, RD, NDI, ODI, and ISOVF were analyzed using TBSS in the FSL Randomise software. Firstly, all subjects' FA data were registered to the standard space FMRIB58 FA template through a nonlinear algorithm FNIRT (Jenkinson et al., 2002). Next, the average FA image was generated by aggregating all FA maps in standard space. Subsequently, a white matter skeleton with an FA threshold of 0.3 was created and used to project the FA, MD, AD, RD, NDI, ODI, and ISOVF images for corresponding values. Group comparisons between the SRC group and the control group were conducted using the general linear model, with age, sex, and education years as covariates. Multiple testing corrections for each contrast were performed using threshold‐free cluster enhancement and 5000 permutation testing. Significant voxels were determined by controlling the familywise error (FWE) rate at corrected *p* < 0.05.

#### 2.4.3 ROI‐based analysis

Sixteen fasciculi from the Johns Hopkins University white matter tractography Atlas were designated as ROIs, specifically including the bilateral anterior thalamic radiations (ATR), corticospinal tract (CST), cingulum, inferior frontal–occipital fasciculus (IFOF), inferior longitudinal fasciculus (ILF), superior longitudinal fasciculus (SLF), uncinate fasciculus (UF), forceps major, and forceps minor. The binary mask images of ROIs were registered onto the individual skeletonized maps, and mean FA, MD, AD, RD, NDI, ODI, and ISOVF values were extracted from each skeletonized ROI. An independent samples *t*‐test was utilized to compare the differences in DTI and NODDI measures between the SRC and HCs groups within 16 main fasciculi. Multiple comparisons were conducted using a false discovery rate (FDR) at *p* < 0.05.

#### 2.4.4 DTI‐ALPS processing

Axial quantitative susceptibility mapping (QSM) images at the lateral ventricle level can clearly show parenchymal vessels run laterally. Using the DTI‐ALPS method to calculate the diffusivities of projected fibers and associated fibers in sections at the lateral ventricle body level, which could indirectly reflect diffusivities along the perivascular space. After generating a color‐coded FA map from DTI measurements and co‐registering it with the ICBM DTI‐81 Atlas, we identified two ROIs around the periventricular area: projection fibers (superior and posterior corona radiata, *z*‐axis, and blue) and association fibers (SLF, *y*‐axis, and green). Subsequently, diffusivities were measured along the *x*‐axis and the *y*‐axis of ROIs within projection fibers (Dxproj, Dyproj), as well as along the *x*‐axis and the *z*‐axis of ROIs within association fibers (Dxassoc, Dzassoc). Following Taoka's description (Taoka et al., 2017), the DTI‐ALPS index was computed by dividing the mean values of Dxproj and Dxassoc by the mean values of Dyproj and Dzaccoc. Statistical significance was determined using FDR correction at *p* < 0.05. The flowchart of this research method is depicted in detail in Supplementary Figure S2.

### 2.5 Statistical analysis

The demographic and MRI measures of all participants were analyzed using SPSS version 22.0. Gender composition differences between groups were assessed using the chi‐square test. The normal distribution of quantitative variables was represented by mean ± standard deviation (SD), and the two‐tailed two‐sample *t*‐test was employed to evaluate differences between the two groups in terms of clinical features, neuropsychological tests, and MRI measures. For non‐normally distributed quantitative variables, median and interquartile range (IQR) were used for representation, while the Mann–Whitney *U* test was utilized. The Spearman correlation coefficient was utilized to investigate the associations among MRI measurements, neuropsychological scores, and clinical characteristics that exhibited significant intragroup differences. Generalized linear mixed models were used to explore the association between clinical assessments of SRC and NODDI measurements. Statistical significance was set at a threshold of *p* < 0.05.

## 3 RESULTS

### 3.1 Participant characteristics

The demographic and clinical characteristics of all participants are summarized in Table 1 and Supplementary Table S1. There were no significant differences between the SRC group and the HCs group in terms of age and years of education. In the SRC group, the average duration of sports participation was 6 years (SD ± 2.8), with a median number of knockouts at 1 (range: 0–15) and a median time since the last SRC at 14 days (IQR: 9–54). The SRC group exhibited comparatively poorer performance in terms of attention, information processing speed, and language learning ability when compared to the HCs group. There were no significant disparities observed in depression and anxiety scores between the two groups.

**TABLE 1:** Demographic and clinical characteristics of all participants.

Characteristic	SRC (*n* = 30)	HCs (*n* = 30)	*p‐*value
Age, years, mean (SD)	20.57 (2.90)	21.93 (4.23)	0.15^a^
Sex, male, *n* (%)	19 (63.3%)	12 (40%)	0.12^b^
Education, years, mean (SD)	10.70 (2.56)	11.70 (3.22)	0.19^a^
Duration in sports, years, mean (SD)	6 (2.8)	NA	NA
Number of knockouts, median (range)	1 (0–15)	NA	NA
Time since the last SRC, days, median (IQR)	14 (9–54)	NA	NA
PCS, mean (SD)	12.3 (10.9)	NA	NA
MoCA, mean (SD)	24.9 (2.5)	26.73 (1.14)	0.001^a^
HAMA, mean (SD)	5.3 (3.8)	4.3 (2.8)	0.26^a^
HAMD, mean (SD)	7.06 (5.15)	5.36 (3.67)	0.15^a^
RAVLT (item 1–5), mean (SD)	43.77 (8.47)	49.03 (10.02)	0.032^a^
Trail making test A, *s*, mean (SD)	31.73 (8.51)	25.47 (8.01)	0.005^a^
Trail making test B, *s*, mean (SD)	77.70 (21.5)	47.16 (22.89)	<0.001^a^
Trail making test B‐A, *s*, mean (SD)	42.0 (22.78)	21.70 (20.86)	0.001^a^
Forward digit span test, mean (SD)	7.37 (1.63)	7.30 (1.20)	0.86^a^
Backward digit span test, mean (SD)	4.97 (1.47)	5.97 (1.96)	0.029^a^

Abbreviations: HAMA, Hamilton Anxiety Scale; HAMD, Hamilton Depression Scale; HCs, Health Controls; IQR, interquartile range; MoCA, Montreal Cognitive Assessment; NA, not available; PCS, Post‐Concussion Scale; RAVLT, Rey Auditory Verbal Learning Test; SD, standard deviations; SRC, Sports‐Related Concussion.

^a^ Two‐sample *t*‐test.

^b^ Chi‐square test.

### 3.2 Differences in DTI metrics

**FIGURE 1:**
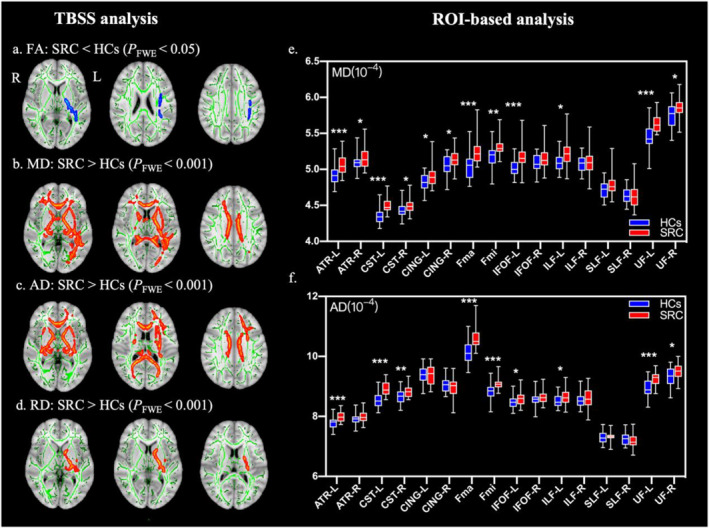
TBSS analysis and ROI‐based analysis in DTI metrics. (A–D) Comparison between the SRC group and the HCs group based on TBSS analysis. Red represents the SRC group voxels with significantly higher values than the HCs group. Blue represents the SRC group voxels with significantly lower values than the HCs group. Green denotes the mean FA skeleton. FA, fractional anisotropy; MD, mean diffusivity; AD, axial diffusivity; and RD, radial diffusivity. (E–F) Comparison between the SRC group and the HCs group based on ROI analysis. The box plots depict the range of data, with the median represented by a solid line in the middle of each box and the quartile range indicated by the outlines of each box. ATR, anterior thalamic radiations; CING, cingulum; CST, corticospinal tract; Fma, forceps major; Fmi, forceps minor; IFOF, inferior frontal–occipital fasciculus; ILF, inferior longitudinal fasciculus; L, left; R, right; ROI, region of interest; SLF, superior longitudinal fasciculus; TBSS, Tract‐based spatial statistics; UF, uncinate fasciculus. * Indicates FDR‐adjusted *p* value < 0.05, ** indicates FDR‐adjusted *p* value < 0.01, and *** indicates FDR‐adjusted *p* value < 0.001.

The TBSS analysis showed significantly lower FA values in the SRC group (*P*_FWE_ < 0.05) (Figure 1A), while MD, AD, and RD values were significantly higher (*P*_FWE_ < 0.001) than the HCs group (Figure 1B–D). The ROI‐based analysis demonstrated lower FA in the right cingulum and left inferior frontal–occipital fasciculus (IFOF), as well as higher RD in the left IFOF, left anterior thalamic radiations (ATR), and bilateral cingulum (Figure S3A,B). Additionally, apart from the left IFOF and ATR, the SRC group exhibited higher MD and AD in the bilateral corticospinal tract (CST), UF, forceps major, and forceps minor (Figure 1E–F).

### 3.3 Differences in NODDI metrics

**FIGURE 2:**
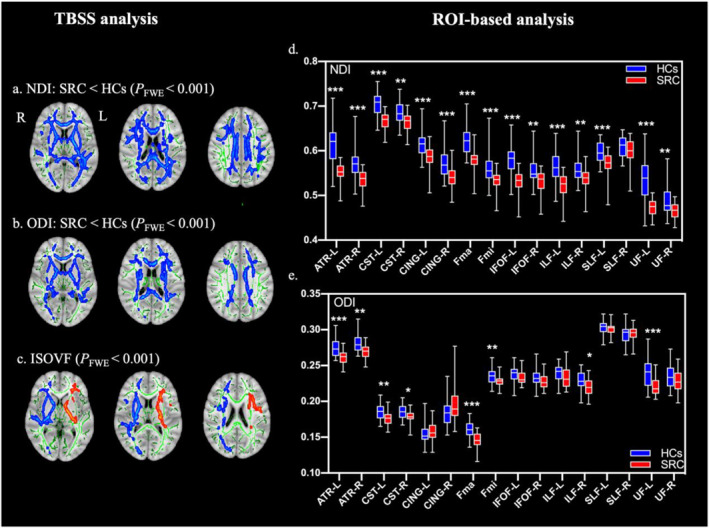
TBSS analysis and ROI‐based analysis in NODDI metrics. (A–C) Comparison between the SRC group and the HCs group based on TBSS analysis. Red represents the SRC group voxels with significantly higher values than the HCs group. Blue represents the SRC group voxels with significantly lower values than the HCs group. Green denotes the mean FA skeleton. NDI, neurite density index; ODI, orientation dispersion index; ISOVF, and orientation dispersion index. (D–E) Comparison between the SRC group and the HCs group based on ROI analysis. The box plots depict the range of data, with the median represented by a solid line in the middle of each box and the quartile range indicated by the outlines of each box. ATR, anterior thalamic radiations; CING, cingulum; CST, corticospinal tract; Fma, forceps major; Fmi, forceps minor; IFOF, inferior frontal–occipital fasciculus; ILF, inferior longitudinal fasciculus; L, left; R, right; SLF, superior longitudinal fasciculus; UF, uncinate fasciculus. * Indicates FDR‐adjusted *p* value < 0.05, ** indicates FDR‐adjusted *p* value < 0.01, and *** indicates FDR‐adjusted *p* value < 0.001.

Compared to the HCs group, the SRC group exhibited significantly lower NDI and ODI in the deep white matter surrounding the lateral ventricle, as well as decreased ISOVF in the right cerebral hemisphere and increased ISOVF in the left cerebral hemisphere (*P*_FWE_ < 0.001) (Figure 2A–C). ROI‐based analysis showed lower NDI across all 15 fasciculi and lower ODI in bilateral ATR, CST, forceps major, forceps minor, and left UF (Figure 2D,E). The left IFOF, ILF, and UF of the SRC group exhibited higher values for ISOVF, while the right IFOF, ILF, and UF showed lower values (Figure S3C). Supplementary Tables S2–S8 present the DTI and NODDI metrics' values for anatomical regions with significant between‐group differences. In terms of NDI, compared to other metrics of NODDI and DTI, a greater degree of significance difference between groups was observed at both the voxel level and the ROI level.

The average values of FA, MD, AD, RD, NDI, ODI, and ISOVF were extracted from each skeletonized ROI. Except for FA and ISOVF, significant changes were observed in the mean values of MD (*p* = 0.001), AD (*p* < 0.001), RD (*p* = 0.026), NDI (*p* < 0.001), and ODI (*p* < 0.001) between the SRC group and the HCs group (Supplementary Table S9).

### 3.4 Differences in the DTI‐ALPS index

**FIGURE 3:**
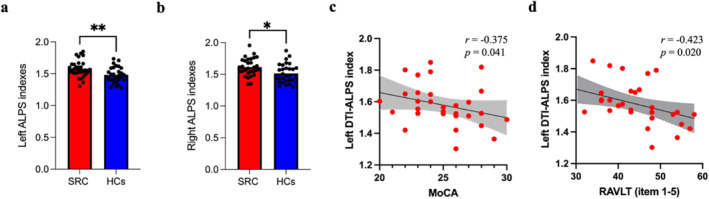
Differences between the SRC group and the HCs group in the ALPS index in bilateral cerebral hemispheres and its correlation with cognitive scales. (A–B) The ALPS index in the SRC group compared to the HCs group. The SRC group showed a higher ALPS index than the HCs group. * Indicates FDR‐adjusted *p* value < 0.05, ** indicates FDR‐adjusted *p* value < 0.01. (c) The left ALPS index was negatively correlated with MoCA scores in the SRC group. (d) The left ALPS index was negatively correlated with RAVLT scores in the SRC group.

The SRC group demonstrated significantly higher left ALPS index and right ALPS index compared to the HCs group (1.58 vs. 1.48, *P*_FDR_ = 0.009, 1.61 vs. 1.51, and *P*_FDR_ = 0.02) (Figure 3A,B). Supplementary Table S10 presents Dxproj, Dxassoc, Dyproj, and Dzaccoc measures for both the left and right hemispheres along with DTI‐ALPS index measures. The Dxassoc values exhibited a significant increase in the SRC group compared to the HCs group. Furthermore, there was a negative correlation observed between the left ALPS index and MoCA scores (*r* = −0.375 and *p* = 0.041) as well as RAVLT scores (*r* = −0.423 and *p* = 0.02) within the SRC group (Figure 3C,D).

### 3.5 Clinical assessments predict NODDI measurement changes

Generalized linear mixed models were employed with ALPS‐L, ALPS‐R, MD, AD, RD, NDI, and ODI as dependent variables. Independent variables included clinical assessments such as duration in sports, number of knockouts, emotion, and cognitive measures acting as predictors. Clinical assessments for boxers of SRC showed associations with NODDI measurements. The number of knockouts was found to be the most reliable clinical predictor for white matter change following SRC. Specifically, a higher number of knockouts was associated with increased MD and RD (*p* = 0.03 and *p* = 0.04), as well as decreased NDI (*p* = 0.03). Furthermore, the forward digit span test emerged as the best predictor for white matter ODI change, where lower scores were correlated with lower ODI values (*p* = 0.015). Additionally, RAVLT's language learning ability score demonstrated predictive value for left ALPS value; lower scores were associated with higher ALPS values (*p* = 0.007) (Supplementary Table S11).

## 4 DISCUSSION

The pathophysiologic effects experienced by active young boxers throughout their careers, including recurrent concussions and sub‐concussions, white matter development and injury, acute and chronic injuries, as well as recovery, have garnered significant attention. However, our current understanding of the pathophysiology of SRCs remains limited, with clinical diagnosis and prognostic judgments primarily relying on the self‐reported symptoms provided by the boxer. To the best of our knowledge, this study represents the first investigation combining NODDI and DTI‐ALPS techniques to explore the alterations in white matter and the glymphatic system among active young boxers. Current results provide additional evidence of abnormalities in DTI parameters (reduced FA, increased MD, AD, and RD), as well as reduced NDI and ODI and a higher ALPS index on active young boxers with SRC. Among all metrics examined, NDI was found to be more sensitive in detecting evidence of diffuse white matter microstructural damage caused by SRC, and the DTI‐ALPS index revealed the glymphatic dysfunction.

### 4.1 DTI and NODDI metrics reveal white matter changes

Punches to the head during daily training and competitions for young boxers elicit both linear and rotational acceleration, with hooks, in particular, inducing a higher degree of concussions due to their elevated levels of rotational acceleration (Ohhashi et al., 2002). Following a concussion, the brain experiences acceleration and deceleration forces along with subsequent shearing and stretching of axons, resulting in white matter damage and abnormal diffusion indicators. DTI analysis revealed significantly lower FA and higher MD, AD, and RD in the SRC group than the HCs group. Decreased FA is considered a reliable biomarker of axonal damage, indicating potential axon disorganization or demyelination, while increased MD may suggest disruption of tissue microstructures, axonal swelling, and extracellular edema. Elevated AD and RD could be indicative of cytoskeletal disruption and demyelination. A substantial body of literature on SRC has extensively documented comparable alterations in DTI metrics (Major et al., 2021; Wright et al., 2021), which aligns with the findings of this investigation. The previous DTI studies conducted on other sports, such as adult rugby and Australian football (Major et al., 2021; Zimmerman et al., 2021), only reported one or two abnormal DTI indexes. However, our study demonstrates that all four DTI indexes of boxers exhibit significant abnormalities. This indicates that boxing, being a direct contact sport, inflicts more evident damage to the brain. Consequently, our findings provide further substantiation for incorporating DTI as a routine sequence in concussion diagnosis.

The NDI metrics reflect the density of neurites, while ODI represents their orientation dispersion. The observed decrease in both NDI and ODI suggests that the loss of neurites may lead to alterations in their geometry and result in a more coherent orientation of white matter neurites. Previous studies have also reported similar reductions in both ODI and NDI in individuals with Alzheimer's disease and Parkinson's disease (Kamagata et al., 2016; Slattery et al., 2017), indicating potential neurodegenerative changes. In terms of damage distribution, the reduction in NDI was found to be statistically significant across the 15 fasciculi examined in this study, while the change area of DTI metrics was smaller than that observed for NDI and ODI metrics. Consequently, NODDI demonstrates greater sensitivity than DTI in detecting microstructural damage associated with SRC, whereas NDI exhibits higher sensitivity than DTI in identifying axonal loss within white matter.

The NODDI metrics also revealed that the left cerebral hemisphere region exhibited a decrease in FA, accompanied by higher ISOVF. Conversely, the right cerebral hemisphere displayed lower ISOVF values. It is worth noting that the median time since the last sports‐related concussion (SRC) among the boxers included in this study was 14 days, which aligns with findings from a previous study on mTBI at a 2‐week time point. These observed alterations in FA and ISOVF can be attributed to an increase in free water content, potentially indicative of neuroinflammation (Palacios et al., 2020). Compared to the control group, the ISOVF trend in the right cerebral hemisphere was different from that in the left cerebral hemisphere in the SRC group, because repetitive SRC is often associated with metabolic disorders and persistent pathobiological conditions (Blennow et al., 2012) and needs to be considered with injury and recovery effects together. Additionally, previous research has demonstrated an asymmetry in white matter pathology on the left side among football players (Veksler et al., 2020). The left cerebral hemisphere, which contains thicker axons within the myelin sheath and is more susceptible to enduring damage, exhibits indications of axonal edema, increased free water content, and neuroinflammation (Anderson et al., 1999). Further investigation into the observed ISOVF in this study should be conducted in conjunction with the timing of SRC and its trajectory.

Although this study reproduced the DTI results of previous studies involving concussions in different sports and found evidence of more fiber bundle involvement in boxers by NODDI measures, it is important to note that the three‐compartment model hypothesized by NODDI does not necessarily assign specific brain structures to corresponding compartments, the ‘stick’ compartment may reflect not only the specific axons but also astrocyte axons or water (Kamiya et al., 2020). In addition, diffusion parameters such as NDI and ODI are indirect measurements of tissue characteristics, and the real histopathological characteristics of microstructure can only be inferred, and possible confounding factors need to be carefully considered. Therefore, combining NODDI with other reliable imaging modalities is more conducive to making more specific inferences about the underlying microstructure. In the future, animal experiments should be conducted to further verify the tissue biological characteristics corresponding to these dispersion parameters, to better apply NODDI technology to the clinic.

### 4.2 DTI‐ALPS index revealed the change in glymphatic function

This study revealed that young boxers who experienced repeated concussions exhibited heightened activity in the glymphatic system, as evidenced by an elevated DTI‐ALPS index. Previous animal studies have demonstrated a compensatory upregulation of aquaporin‐4 (AQP4) expression throughout the entire brain following traumatic injury (Higashida et al., 2011; Ren et al., 2013). The involvement of AQP4 in the transport of cerebrospinal fluid and interstitial fluid, as well as its crucial role in waste removal from the glymphatic system throughout the brain, has been highlighted (Mestre et al., 2018). Following brain injury, mice with a knockout of the AQP4 gene exhibited exacerbated dysfunction within the glymphatic pathway (Iliff et al., 2014). Additionally, the glymphatic system is influenced by cerebral blood perfusion and vascular pulsation. A study conducted on adolescent female rats with repetitive mTBI revealed an increase in glymphatic system influx but a decrease in efflux, potentially indicating an enhanced glymphatic flow to accommodate the detrimental effects of brain injury (Christensen et al., 2020). Restoring brain damage caused by SRC and reducing secondary damage may require a more active glymphatic system. A study examining mTBI across a wide age range (15–90 years) also confirmed heightened functional activity of the glymphatic system, with effects being more pronounced in younger individuals (Dai et al., 2023). It is important to note that our findings are specific to mTBI and may differ in trauma groups with varying injury severity or animal models with different etiologies (Li et al., 2020; J. H. Park et al., 2022). The focus of this study lies on young boxers with recurrent SRC, making them a suitable representative sample for this cohort. However, it is important to note that the utilization of DTI‐ALPS technology for assessing glymphatic system activity still necessitates rigorous pathophysiological validation in future investigations.

The study revealed a negative correlation between higher DTI‐ALPS indexes and specific cognitive scales. Previous animal experiments suggest that increased glymphatic influx and reduced efflux after brain trauma may clear inflammatory molecules important for injury recovery and increase brain glucose and glutamate levels, leading to dysfunction and cell death (Christensen et al., 2020). The function of the glymphatic system must be maintained in equilibrium, as insufficient or excessive clearance can result in maladaptation and adverse consequences for the body (Li et al., 2020). It is imperative to extend the findings of this study conducted on young boxers to elderly or retired boxers, to ascertain whether the current changes represent compensatory mechanisms or an early pathologically active state of neurodegenerative disease.

### 4.3 Associations between clinical assessments and NODDI measurements

The areas of white matter change in this study were primarily localized in the periventricular deep white matter regions bilaterally. These white matter tracts are anatomically adjacent to the brain's central core and consist of elongated axonal fibers, making them more susceptible to shear and strain forces (Arbogast & Margulies, 1998). These deep periventricular white matter tracts play a crucial role in the large‐scale structural network, contributing significantly to neural connectivity within the brain. Structural damage disrupts these intricate neural connections, thereby directly impacting cognitive function. This phenomenon may elucidate the diminished attentional capacity, cognitive abilities, and language learning ability observed in boxers, which exhibit a correlation with certain NODDI metrics. Furthermore, our findings indicate that an increased mean MD and decreased mean NDI in the SRC group are positively associated with the frequency of knockouts. The number of head impacts is the most reliable clinical predictor for white matter changes following sports‐related concussions (SRC), indicating that an increased frequency of head injuries leads to a greater loss of neuronal axons. Previous studies on boxers and mixed martial artists have also demonstrated a strong correlation between the number of knockouts and longitudinal diffusivity as well as transversal diffusivity (Shin et al., 2014). Collectively, our study findings support the cumulative impact of repetitive SRC and its exacerbation of white matter damage.

This study has several limitations. Firstly, the quantification of definite concussions and sub‐concussions proves challenging due to the absence of specific clinical diagnostic criteria. We solely relied on self‐reported knockouts and details provided by the boxer regarding their most recent concussion, which may have led to an underestimation of sustained concussions and compromised measurement accuracy. Secondly, the study focused on a limited sample of young boxers. To validate the results of this study and minimize errors, it is better to include a larger number of young boxers in future research. Additionally, subgroup comparisons and longitudinal follow‐up studies should involve athletes from various age groups to gain comprehensive insights into the progression of white matter changes and glymphatic dysfunction at different stages of development. Another limitation of the study is that there was an imbalance in the sex ratio between the two groups, with a higher proportion of males in the SRC group. Moreover, this study did not incorporate white matter indicators to assess the brain's connectivity and topological alterations in the whole‐brain connectome, which are essential for elucidating higher‐order cognitive functions. Lastly, it is crucial to conduct additional animal experiments to validate the detrimental effects of hyperactive brain glymphatic system function in young individuals with SRC.

## 5 CONCLUSION

The NODDI‐based study revealed evidence of diffuse white matter damage in young boxers with SRC and identified regions where DTI indicators showed no abnormalities, indicating the sensitivity of NODDI metrics in detecting pathological changes in white matter microstructure. Furthermore, this study demonstrated the impact of repeated concussion and sub‐concussion on the glymphatic function of young boxers using the DTI‐ALPS method. Importantly, despite the youthfulness of the athletes, there have been notable neurological alterations observed in the active boxers' brains, underscoring the necessity for long‐term monitoring and preservation of their neurological well‐being.

## AUTHOR CONTRIBUTIONS

**Wenjing Huang**: Study concept and design, data analyses and statistical analyses, and drafting manuscript and interpretation of data and results. **Laiyang Ma**: Data analyses, and manuscript preparation. **Jiahao Yan**: Data acquisition. **Wanjun Hu**: Data analyses and interpretation of data. **Guangyao Liu:** Study concept and design. **Rui Wang**: Data acquisition. **Jing Zhang**: Study concept and design, interpretation of data, and critical revision of the manuscript for important intellectual content.

## CONFLICT OF INTEREST STATEMENT

No conflict of interest to disclose.

## Supporting information

Supporting Information S1
