# Supplementary material for: Neurite orientation dispersion and density imaging reveals abnormal white matter and glymphatic function in active young boxers
Source: Eur J Sport Sci. 2024 Apr 26;24(7):975–86. doi: 10.1002/ejsc.12113 (PMC11235717; doi:10.1002/ejsc.12113)
Supplement: Supplementary file 1 — Supporting Information S1 [file EJSC-24-975-s001.docx]

**Supplementary Work:**

**Supplementary Figure S1: ﻿Flow diagram of the inclusion process.**

**
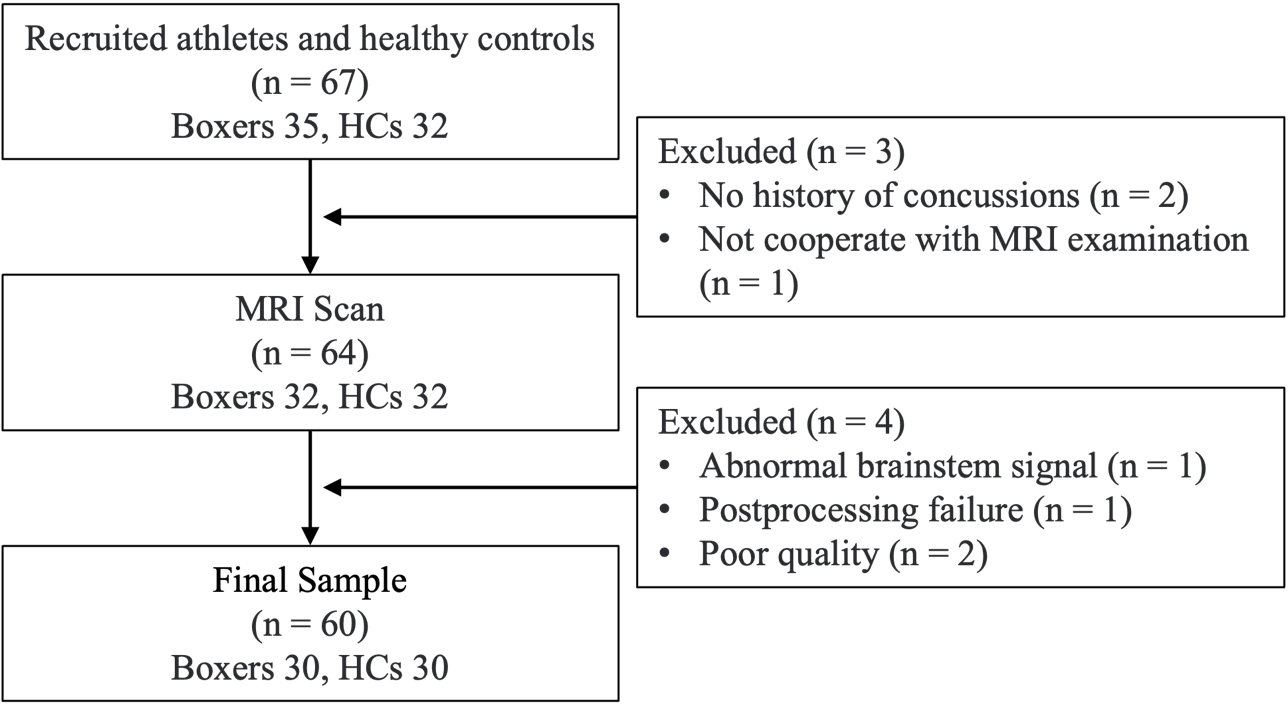
**

**﻿****Supplementary Table S1: Demographic information and clinical characteristics of boxers.**

| No. | gender | Age  (years) | Education  (years) | Duration in sports  (years) | Number of knockouts | Time since the last SRC (days) | Location of the last SRC | Symptoms of the last SRC |
| --- | --- | --- | --- | --- | --- | --- | --- | --- |
| SRC002 | M | 17 | 9 | 5 | 8 | 12 | lower jaw | LOC (8s) & headache (3d) |
| SRC003 | M | 18 | 9 | 5 | 2 | 23 | occipital | headache (1d) |
| SRC004 | M | 18 | 9 | 3 | 1 | 90 | left temporal | LOC (6s) & headache (2d) |
| SRC005 | M | 25 | 15 | 11 | 9 | 90 | lower jaw | LOC (60s) & amnesia (1h) |
| SRC006 | M | 20 | 5 | 8 | 1 | 60 | occipital | LOC (3s) |
| SRC007 | M | 24 | 5 | 10 | 5 | 52 | lower jaw | LOC (3s) & amnesia (5h) |
| SRC008 | M | 18 | 10 | 5 | 1 | 22 | forehead | None |
| SRC009 | M | 20 | 9 | 4 | 1 | 60 | lower jaw | None |
| SRC010 | M | 22 | 10 | 7 | 3 | 32 | lower jaw | LOC (2s) |
| SRC011 | F | 21 | 11 | 4 | 0 | 7 | left temporal | LOC (2s) & headache (2d) |
| SRC012 | F | 22 | 12 | 9 | 0 | 7 | forehead | None |
| SRC013 | F | 20 | 9 | 4 | 0 | 9 | left temporal | headache (3d) |
| SRC014 | M | 25 | 12 | 6 | 1 | 9 | left parietal | LOC (2s) |
| SRC015 | M | 26 | 15 | 9 | 1 | 11 | forehead | LOC (15s) |
| SRC016 | M | 25 | 12 | 7 | 0 | 40 | forehead | None |
| SRC017 | M | 21 | 7 | 8 | 3 | 90 | right temporal | LOC (3s) & amnesia (3h) |
| No. | gender | Age  (years) | Education  (years) | Duration in sports  (years) | Number of knockouts | Time since the last SRC (days) | Location of the last SRC | Symptoms of the last SRC |
| SRC018 | F | 21 | 12 | 6 | 1 | 14 | forehead | None |
| SRC019 | F | 18 | 8 | 2 | 1 | 14 | right temporal | LOC (60s) & headache (1d) |
| SRC020 | F | 18 | 8 | 3 | 2 | 12 | left temporal | LOC (3s) |
| SRC021 | F | 19 | 12 | 5 | 15 | 7 | forehead | LOC (3s) |
| SRC022 | M | 18 | 11 | 1 | 1 | 10 | forehead | LOC (60s) & headache (1d) |
| SRC023 | M | 17 | 13 | 1 | 0 | 10 | forehead | LOC (1s) |
| SRC024 | F | 23 | 12 | 10 | 1 | 60 | lower jaw | LOC (2s) |
| SRC025 | F | 20 | 12 | 10 | 1 | 7 | occipital | None |
| SRC026 | F | 24 | 15 | 8 | 1 | 11 | left temporal | LOC (1200s) & headache (3d) |
| SRC027 | F | 17 | 12 | 4 | 1 | 7 | occipital | LOC (3s) |
| SRC028 | M | 25 | 13 | 10 | 0 | 7 | forehead | None |
| SRC029 | M | 20 | 12 | 6 | 10 | 60 | right temporal | LOC (3s) |
| SRC030 | M | 18 | 11 | 4 | 1 | 18 | right temporal | LOC (240s) & headache (2h) |
| SRC031 | M | 17 | 11 | 5 | 2 | 50 | left temporal | LOC (2s) & headache (2d) |

M: male; F: female; cm: centimeter; kg: kilogram; SRC: sports-related concussion; LOC: Loss of consciousness; s: second; h: hour; d: day.

**Supplementary Figure S2: ﻿Overview of the study methods.**


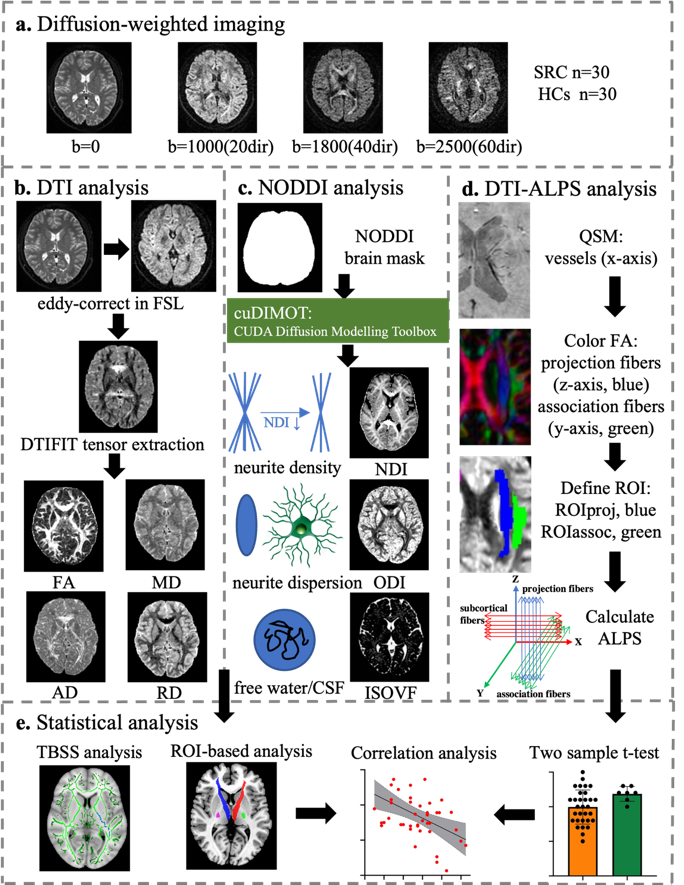


(a) The NODDI protocol. (b) DTI data preprocessing. (c) NODDI data preprocessing. (d) DTI-ALPS analysis. (e) Statistical analysis. HCs = healthy controls; SRC = sport-related concussion; DTI = diffusion tensor imaging; DTI along perivascular spaces = DTI-ALPS; NODDI = neurite orientation dispersion and density imaging; FA = fractional anisotropy; MD = mean diffusivity; AD = axial diffusivity; RD = radial diffusivity; NDI = neurite density index; ODI = orientation dispersion index; ISOVF = isotropic volume fraction; QSM = quantitative susceptibility mapping; ROI = region of interest.

**Supplementary Figure S3: ROI-based group comparison between HCs and athletes with SRC in ISOVF.**

**
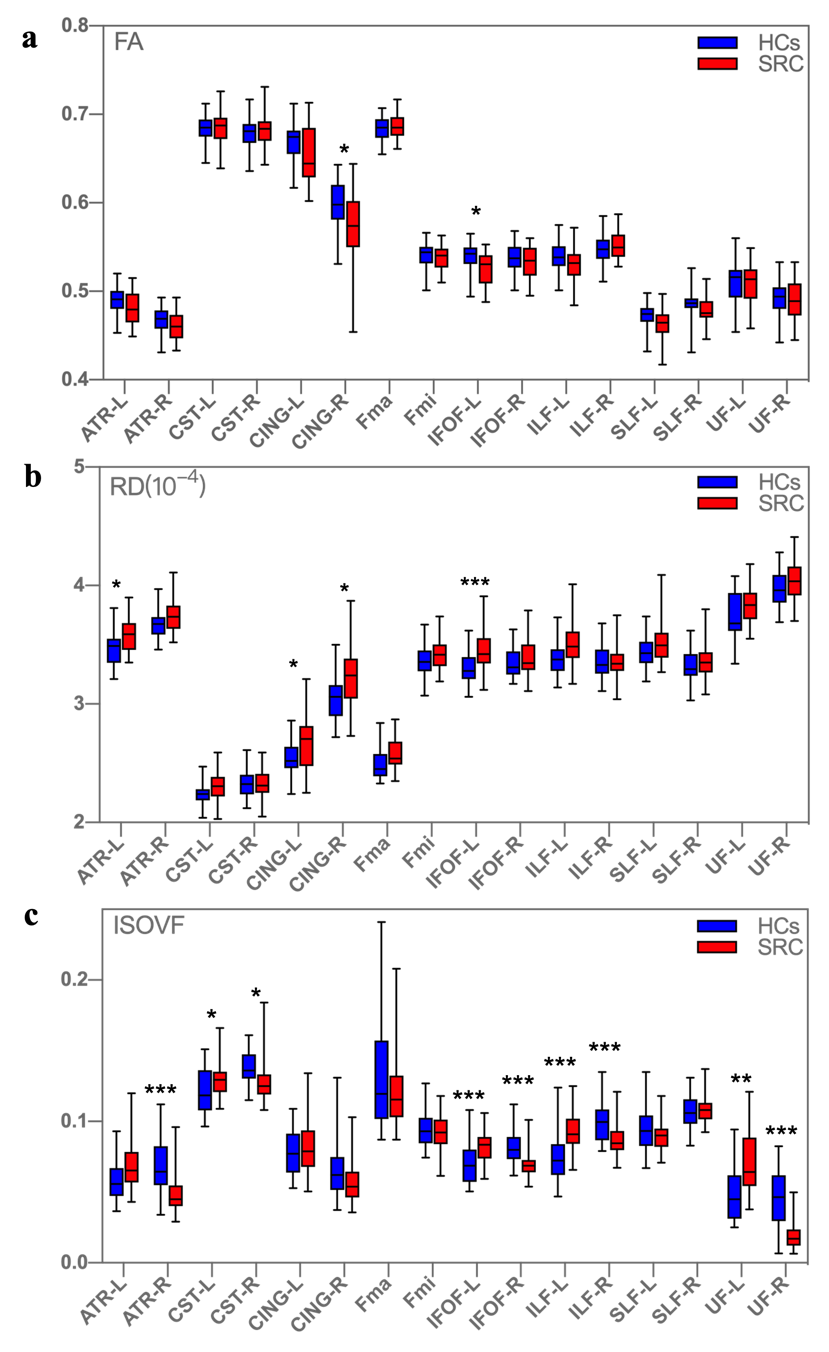
**

* Indicates FDR-adjusted *P* value < 0.05, ** indicates FDR-adjusted *P* value < 0.01, *** indicates FDR-adjusted *P* value < 0.001. ATR = anterior thalamic radiations; CST = cortico-spinal tract; CING = cingulum; Fma = forceps major; Fmi = forceps minor; IFOF = inferior frontal-occipital fasciculus; ILF = inferior longitudinal fasciculus; SLF = superior longitudinal fasciculus; UF = uncinate fasciculus; L = left; R = right.

**Supplementary Table S2:** **Anatomical regions that showed significant between-group differences in FA**

| White Matter ROIs | Abbreviations | FA-HCs(n=30) | FA-SRC(n=30) | t-value | FA | *P_FDR_-* |
| --- | --- | --- | --- | --- | --- | --- |
|  |  | Mean±SD | Mean±SD |  | *P*-value | *adjusted* |
| Anterior.thalamic.radiation.L | ATR-L | 0.49±0.02 | 0.48±0.02 | -1.64 | 0.108 | 0.238 |
| Anterior.thalamic.radiation.R | ATR-R | 0.47±0.02 | 0.46±0.02 | -1.58 | 0.119 | 0.238 |
| Corticospinal.tract.L | CST-L | 0.68±0.01 | 0.68±0.02 | 0.84 | 0.933 | 0.933 |
| Corticospinal.tract.R | CST-R | 0.68±0.02 | 0.68±0.02 | 0.61 | 0.544 | 0.682 |
| Cingulum.(cingulate.gyrus).L | CING-L | 0.67±0.02 | 0.65±0.03 | -2.52 | 0.015* | 0.080 |
| Cingulum.(cingulate.gyrus).R | CING-R | 0.60±0.03 | 0.57±0.04 | -3.26 | 0.002** | 0.016* |
| Forceps.major | Fma | 0.68±0.01 | 0.69±0.019 | 0.60 | 0.554 | 0.682 |
| Forceps.minor | Fmi | 0.54±0.01 | 0.54±0.01 | -0.61 | 0.543 | 0.682 |
| Inferior.fronto-occipital.fasciculus.L | IFOF-L | 0.54±0.01 | 0.53±0.02 | -3.19 | 0.002** | 0.016* |
| Inferior.fronto-occipital.fasciculus.R | IFOF-R | 0.54±0.02 | 0.53±0.02 | -0.94 | 0.354 | 0.629 |
| Inferior.longitudinal.fasciculus.L | ILF-L | 0.54±0.02 | 0.53±0.02 | -1.81 | 0.075 | 0.2 |
| Inferior.longitudinal.fasciculus.R | ILF-R | 0.55±0.02 | 0.55±0.02 | 0.80 | 0.428 | 0.682 |
| Superior.longitudinal.fasciculus.L | SLF-L | 0.47±0.01 | 0.46±0.02 | -2.38 | 0.021* | 0.084 |
| Superior.longitudinal.fasciculus.R | SLF-R | 0.49±0.02 | 0.48±0.02 | -1.86 | 0.069 | 0.2 |
| Uncinate.fasciculus.L | UF-L | 0.51±0.03 | 0.51±0.03 | -0.20 | 0.841 | 0.897 |
| Uncinate.fasciculus.R | UF-R | 0.49±0.02 | 0.49±0.02 | -0.37 | 0.715 | 0.812 |

ROI: region-of-interest; SRC: sports-related concussion; HCs: healthy controls; SD: standard deviation; FA: fractional anisotropy; L: left; R: right

* indicates *P* value < 0.05, ** indicates *P* value < 0.01.

**Supplementary Table S3: Anatomical regions that showed significant between-group differences in MD**

| White Matter ROIs | Abbreviations | MD-HCs(n=30) | MD-SRC(n=30) | t-value | MD | *P_FDR_-* |
| --- | --- | --- | --- | --- | --- | --- |
|  |  | Mean±SD(×10^-4^) | Mean±SD(×10^-4^) |  | *P*-value | *adjusted* |
| Anterior.thalamic.radiation.L | ATR-L | 4.91±0.14 | 5.06±0.13 | 4.17 | ＜0.001*** | ＜0.001*** |
| Anterior.thalamic.radiation.R | ATR-R | 5.09±0.12 | 5.16±0.14 | 2.14 | 0.037* | 0.049* |
| Corticospinal.tract.L | CST-L | 4.35±0.12 | 4.50±0.11 | 5.30 | ＜0.001*** | ＜0.001*** |
| Corticospinal.tract.R | CST-R | 4.43±0.11 | 4.50±0.11 | 2.34 | 0.023* | 0.034* |
| Cingulum.(cingulate.gyrus).L | CING-L | 4.82±0.12 | 4.91±0.15 | 2.58 | 0.012* | 0.021* |
| Cingulum.(cingulate.gyrus).R | CING-R | 5.05±0.14 | 5.14±0.13 | 2.33 | 0.023* | 0.034* |
| Forceps.major | Fma | 5.05±0.19 | 5.25±0.17 | 4.35 | ＜0.001*** | ＜0.001*** |
| Forceps.minor | Fmi | 5.18±0.17 | 5.31±0.14 | 3.37 | 0.001** | 0.003** |
| Inferior.fronto-occipital.fasciculus.L | IFOF-L | 5.02±0.13 | 5.17±0.17 | 3.95 | ＜0.001*** | ＜0.001*** |
| Inferior.fronto-occipital.fasciculus.R | IFOF-R | 5.09±0.12 | 5.15±0.15 | 1.74 | 0.087 | 0.099 |
| Inferior.longitudinal.fasciculus.L | ILF-L | 5.10±0.14 | 5.22±0.18 | 2.89 | 0.005** | 0.011* |
| Inferior.longitudinal.fasciculus.R | ILF-R | 5.08±0.12 | 5.11±0.18 | 0.63 | 0.531 | 0.566 |
| Superior.longitudinal.fasciculus.L | SLF-L | 4.72±0.12 | 4.79±0.15 | 1.97 | 0.054 | 0.067 |
| Superior.longitudinal.fasciculus.R | SLF-R | 4.63±0.11 | 4.63±0.15 | 0.14 | 0.888 | 0.888 |
| Uncinate.fasciculus.L | UF-L | 5.47±0.20 | 5.64±0.12 | 4.12 | ＜0.001*** | ＜0.001*** |
| Uncinate.fasciculus.R | UF-R | 5.75±0.17 | 5.86±0.14 | 2.80 | 0.007** | 0.014* |

ROI: region-of-interest; SRC: sports-related concussion; HCs: healthy controls; SD: standard deviation; MD: mean diffusivity; L: left; R: right

* indicates *P* value < 0.05, ** indicates *P* value < 0.01, *** indicates *P* value *P* < 0.001.

**Supplementary Table S4:** **Anatomical regions that showed significant between-group differences in AD**

| White Matter ROIs | Abbreviations | AD-HCs(n=30) | AD-SRC(n=30) | t-value | AD | *P_FDR_-* |
| --- | --- | --- | --- | --- | --- | --- |
|  |  | Mean±SD(×10^-4^) | Mean±SD(×10^-4^) |  | *P*-value | *adjusted* |
| Anterior.thalamic.radiation.L | ATR-L | 7.78±0.21 | 8.00±0.17 | 4.39 | ＜0.001*** | ＜0.001*** |
| Anterior.thalamic.radiation.R | ATR-R | 7.90±0.17 | 8.00±0.19 | 2.00 | 0.050 | 0.08 |
| Corticospinal.tract.L | CST-L | 8.57±0.31 | 8.91±0.23 | 4.90 | ＜0.001*** | ＜0.001*** |
| Corticospinal.tract.R | CST-R | 8.67±0.27 | 8.86±0.23 | 3.14 | 0.003** | 0.008** |
| Cingulum.(cingulate.gyrus).L | CING-L | 9.37±0.32 | 9.38±0.33 | 0.08 | 0.933 | 0.933 |
| Cingulum.(cingulate.gyrus).R | CING-R | 9.08±0.27 | 8.96±0.36 | 1.43 | 0.159 | 0.196 |
| Forceps.major | Fma | 10.15±0.40 | 10.60±0.36 | 4.65 | ＜0.001*** | ＜0.001*** |
| Forceps.minor | Fmi | 8.83±0.31 | 9.09±0.19 | 3.86 | ＜0.001*** | ＜0.001*** |
| Inferior.fronto-occipital.fasciculus.L | IFOF-L | 8.47±0.19 | 8.61±0.23 | 2.62 | 0.011* | 0.024* |
| Inferior.fronto-occipital.fasciculus.R | IFOF-R | 8.56±0.19 | 8.65±0.23 | 1.77 | 0.082 | 0.120 |
| Inferior.longitudinal.fasciculus.L | ILF-L | 8.52±0.22 | 8.66±0.27 | 2.28 | 0.026* | 0.046* |
| Inferior.longitudinal.fasciculus.R | ILF-R | 8.53±0.21 | 8.62±0.31 | 1.31 | 0.195 | 0.223 |
| Superior.longitudinal.fasciculus.L | SLF-L | 7.30±0.19 | 7.33±0.17 | 0.68 | 0.498 | 0.531 |
| Superior.longitudinal.fasciculus.R | SLF-R | 7.25±0.20 | 7.16±0.22 | 1.65 | 0.104 | 0.139 |
| Uncinate.fasciculus.L | UF-L | 8.93±0.33 | 9.25±0.25 | 4.23 | ＜0.001*** | ＜0.001*** |
| Uncinate.fasciculus.R | UF-R | 9.30±0.33 | 9.50±0.26 | 2.59 | 0.012* | 0.024* |

ROI: region-of-interest; SRC: sports-related concussion; HCs: healthy controls; SD: standard deviation; AD: axial diffusivity; L: left; R: right

* indicates *P* value < 0.05, ** indicates *P* value < 0.01, *** indicates *P* value *P* < 0.001.

**Supplementary Table S5: Anatomical regions that showed significant between-group differences in RD**

| White Matter ROIs | Abbreviations | RD-HCs(n=30) | RD-SRC(n=30) | t-value | RD | *P_FDR_-* |
| --- | --- | --- | --- | --- | --- | --- |
|  |  | Mean±SD(×10^-4^) | Mean±SD(×10^-4^) |  | *P*-value | *adjusted* |
| Anterior.thalamic.radiation.L | ATR-L | 3.47±0.14 | 3.59±0.15 | 3.02 | 0.004** | 0.021* |
| Anterior.thalamic.radiation.R | ATR-R | 3.68±0.12 | 3.74±0.15 | 1.70 | 0.095 | 0.138 |
| Corticospinal.tract.L | CST-L | 2.24±0.08 | 2.30±0.13 | 2.04 | 0.046 | 0.08 |
| Corticospinal.tract.R | CST-R | 2.32±0.11 | 2.32±0.13 | -0.06 | 0.95 | 0.95 |
| Cingulum.(cingulate.gyrus).L | CING-L | 2.55±0.15 | 2.68±0.22 | 2.75 | 0.008** | 0.032* |
| Cingulum.(cingulate.gyrus).R | CING-R | 3.04±0.18 | 3.22±0.25 | 3.28 | 0.002** | 0.016* |
| Forceps.major | Fma | 2.50±0.14 | 2.57±0.13 | 2.08 | 0.042* | 0.08 |
| Forceps.minor | Fmi | 3.35±0.14 | 3.42±0.13 | 2.01 | 0.050 | 0.08 |
| Inferior.fronto-occipital.fasciculus.L | IFOF-L | 3.30±0.13 | 3.45±0.18 | 3.85 | ＜0.001*** | ＜0.001*** |
| Inferior.fronto-occipital.fasciculus.R | IFOF-R | 3.35±0.13 | 3.40±0.16 | 1.17 | 0.246 | 0.28 |
| Inferior.longitudinal.fasciculus.L | ILF-L | 3.39±0.14 | 3.50±0.19 | 2.45 | 0.018* | 0.05 |
| Inferior.longitudinal.fasciculus.R | ILF-R | 3.36±0.13 | 3.35±0.15 | -0.27 | 0.791 | 0.84 |
| Superior.longitudinal.fasciculus.L | SLF-L | 3.44±0.11 | 3.53±0.17 | 2.41 | 0.019* | 0.05 |
| Superior.longitudinal.fasciculus.R | SLF-R | 3.32±0.12 | 3.36±0.14 | 1.17 | 0.248 | 0.28 |
| Uncinate.fasciculus.L | UF-L | 3.73±0.21 | 3.83±0.15 | 2.08 | 0.042* | 0.08 |
| Uncinate.fasciculus.R | UF-R | 3.96±0.17 | 4.04±018 | 1.50 | 0.139 | 0.19 |

ROI: region-of-interest; SRC: sports-related concussion; HCs: healthy controls; SD: standard deviation; MD: mean diffusivity; L: left; R: right

* indicates *P* value < 0.05, ** indicates *P* value < 0.01, *** indicates *P* value *P* < 0.001.

**Supplementary Table S6: Anatomical regions that showed significant between-group differences in NDI**

| White Matter ROIs | Abbreviations | NDI-HCs(n=30) | NDI-SRC(n=30) | t-value | NDI | *P_FDR_-* |
| --- | --- | --- | --- | --- | --- | --- |
|  |  | Mean±SD | Mean±SD |  | *P*-value | *adjusted* |
| Anterior.thalamic.radiation.L | ATR-L | 0.61±0.05 | 0.55±0.02 | -6.85 | ＜0.001*** | ＜0.001*** |
| Anterior.thalamic.radiation.R | ATR-R | 0.57±0.04 | 0.53±0.02 | -4.84 | ＜0.001*** | ＜0.001*** |
| Corticospinal.tract.L | CST-L | 0.71±0.03 | 0.67±0.02 | -6.27 | ＜0.001*** | ＜0.001*** |
| Corticospinal.tract.R | CST-R | 0.69±0.03 | 0.66±0.02 | -3.65 | 0.001** | 0.001** |
| Cingulum.(cingulate.gyrus).L | CING-L | 0.62±0.03 | 0.59±0.03 | -4.36 | ＜0.001*** | ＜0.001*** |
| Cingulum.(cingulate.gyrus).R | CING-R | 0.57±0.03 | 0.54±0.03 | -4.13 | ＜0.001*** | ＜0.001*** |
| Forceps.major | Fma | 0.62±0.03 | 0.58±0.02 | -5.90 | ＜0.001*** | ＜0.001*** |
| Forceps.minor | Fmi | 0.56±0.04 | 0.53±0.02 | -3.72 | ＜0.001*** | ＜0.001*** |
| Inferior.fronto-occipital.fasciculus.L | IFOF-L | 0.58±0.04 | 0.53±0.03 | -6.09 | ＜0.001*** | ＜0.001*** |
| Inferior.fronto-occipital.fasciculus.R | IFOF-R | 0.56±0.03 | 0.53±0.03 | -3.52 | 0.001** | 0.001** |
| Inferior.longitudinal.fasciculus.L | ILF-L | 0.56±0.04 | 0.52±0.03 | -4.99 | ＜0.001*** | ＜0.001*** |
| Inferior.longitudinal.fasciculus.R | ILF-R | 0.56±0.03 | 0.54±0.03 | -3.01 | 0.004** | 0.004** |
| Superior.longitudinal.fasciculus.L | SLF-L | 0.60±0.02 | 0.578±0.03 | -4.06 | ＜0.001*** | ＜0.001*** |
| Superior.longitudinal.fasciculus.R | SLF-R | 0.61±0.02 | 0.60±0.03 | -1.85 | 0.07 | 0.07 |
| Uncinate.fasciculus.L | UF-L | 0.54±0.05 | 0.47±0.02 | -6.68 | ＜0.001*** | ＜0.001*** |
| Uncinate.fasciculus.R | UF-R | 0.49±0.04 | 0.47±0.02 | -3.06 | 0.003** | 0.003** |

ROI: region-of-interest; SRC: sports-related concussion; HCs: healthy controls; SD: standard deviation; NDI: neurite density index; L: left; R: right

* indicates *P* value < 0.05, ** indicates *P* value < 0.01, *** indicates *P* value *P* < 0.001.

**Supplementary Table S7: Anatomical regions that showed significant between-group differences in ODI**

| White Matter ROIs | Abbreviations | ODI-HCs(n=30) | ODI-SRC(n=30) | t-value | ODI | *P_FDR_-* |
| --- | --- | --- | --- | --- | --- | --- |
|  |  | Mean±SD | Mean±SD |  | *P*-value | *adjusted* |
| Anterior.thalamic.radiation.L | ATR-L | 0.28±0.01 | 0.26±0.01 | -4.43 | ＜0.001*** | ＜0.001*** |
| Anterior.thalamic.radiation.R | ATR-R | 0.28±0.01 | 0.27±0.01 | -3.68 | 0.001** | 0.004** |
| Corticospinal.tract.L | CST-L | 0.19±0.01 | 0.18±0.01 | -3.23 | 0.002** | 0.005** |
| Corticospinal.tract.R | CST-R | 0.19±0.01 | 0.18±0.01 | -2.66 | 0.01* | 0.02* |
| Cingulum.(cingulate.gyrus).L | CING-L | 0.16±0.02 | 0.16±0.02 | 0.28 | 0.783 | 0.783 |
| Cingulum.(cingulate.gyrus).R | CING-R | 0.18±0.02 | 0.20±0.03 | 2.03 | 0.047* | 0.075 |
| Forceps.major | Fma | 0.16±0.01 | 0.15±0.01 | -4.53 | ＜0.001*** | ＜0.001*** |
| Forceps.minor | Fmi | 0.24±0.01 | 0.23±0.01 | -3.31 | 0.002** | 0.005** |
| Inferior.fronto-occipital.fasciculus.L | IFOF-L | 0.24±0.01 | 0.23±0.01 | -1.67 | 0.101 | 0.124 |
| Inferior.fronto-occipital.fasciculus.R | IFOF-R | 0.23±0.01 | 0.23±0.01 | -1.84 | 0.071 | 0.095 |
| Inferior.longitudinal.fasciculus.L | ILF-L | 0.24±0.01 | 0.23±0.01 | -2.16 | 0.035* | 0.062 |
| Inferior.longitudinal.fasciculus.R | ILF-R | 0.23±0.01 | 0.22±0.01 | -2.93 | 0.005** | 0.011* |
| Superior.longitudinal.fasciculus.L | SLF-L | 0.30±0.01 | 0.30±0.01 | -0.60 | 0.548 | 0.626 |
| Superior.longitudinal.fasciculus.R | SLF-R | 0.29±0.01 | 0.29±0.01 | 0.54 | 0.589 | 0.628 |
| Uncinate.fasciculus.L | UF-L | 0.24±0.02 | 0.22±0.01 | -4.43 | ＜0.001*** | ＜0.001*** |
| Uncinate.fasciculus.R | UF-R | 0.24±0.02 | 0.23±0.02 | -1.86 | 0.068 | 0.095 |

ROI: region-of-interest; SRC: sports-related concussion; HCs: healthy controls; SD: standard deviation; ODI: orientation dispersion index; L: left; R: right. * indicates *P* value < 0.05, ** indicates *P* value < 0.01, *** indicates *P* value *P* < 0.001.

**Supplementary Table S8: Anatomical regions that showed significant between-group differences in ISOVF**

| White Matter ROIs | Abbreviations | ISOVF-HCs(n=30) | ISOVF-SRC(n=30) | t-value | ISOVF | *P_FDR_-* |
| --- | --- | --- | --- | --- | --- | --- |
|  |  | Mean±SD | Mean±SD |  | *P*-value | *adjusted* |
| Anterior.thalamic.radiation.L | ATR-L | 0.06±0.02 | 0.07±0.02 | 2.56 | 0.013* | 0.026* |
| Anterior.thalamic.radiation.R | ATR-R | 0.07±0.02 | 0.05±0.01 | -4.63 | ＜0.001*** | ＜0.001*** |
| Corticospinal.tract.L | CST-L | 0.12±0.02 | 0.13±0.01 | 2.29 | 0.026* | 0.042* |
| Corticospinal.tract.R | CST-R | 0.14±0.01 | 0.13±0.02 | -2.38 | 0.021* | 0.037* |
| Cingulum.(cingulate.gyrus).L | CING-L | 0.08±0.02 | 0.08±0.02 | 0.74 | 0.465 | 0.496 |
| Cingulum.(cingulate.gyrus).R | CING-R | 0.06±0.02 | 0.06±0.02 | -1.33 | 0.189 | 0.275 |
| Forceps.major | Fma | 0.14±0.05 | 0.12±0.03 | -0.98 | 0.242 | 0.323 |
| Forceps.minor | Fmi | 0.09±0.01 | 0.09±0.01 | -0.84 | 0.404 | 0.462 |
| Inferior.fronto-occipital.fasciculus.L | IFOF-L | 0.07±0.01 | 0.08±0.01 | 3.96 | ＜0.001*** | ＜0.001*** |
| Inferior.fronto-occipital.fasciculus.R | IFOF-R | 0.08±0.01 | 0.07±0.01 | -4.62 | ＜0.001*** | ＜0.001*** |
| Inferior.longitudinal.fasciculus.L | ILF-L | 0.07±0.02 | 0.09±0.01 | 4.59 | ＜0.001*** | ＜0.001*** |
| Inferior.longitudinal.fasciculus.R | ILF-R | 0.10±0.02 | 0.09±0.01 | -4.19 | ＜0.001*** | ＜0.001*** |
| Superior.longitudinal.fasciculus.L | SLF-L | 0.09±0.02 | 0.09±0.01 | -0.91 | 0.366 | 0.451 |
| Superior.longitudinal.fasciculus.R | SLF-R | 0.11±0.01 | 0.11±0.01 | 0.66 | 0.515 | 0.515 |
| Uncinate.fasciculus.L | UF-L | 0.05±0.02 | 0.07±0.02 | 3.68 | 0.001** | 0.002** |
| Uncinate.fasciculus.R | UF-R | 0.05±0.02 | 0.02±0.01 | -7.02 | ＜0.001*** | ＜0.001*** |

ROI: region-of-interest; SRC: sports-related concussion; HCs: healthy controls; SD: standard deviation; ISOVF: isotropic volume fraction; L: left; R: right; * indicates *P* value < 0.05, ** indicates *P* value < 0.01, *** indicates *P* value *P* < 0.001.

**Supplementary Table S9: Mean NODDI measures of the SRC group and HCs group**

|  | SRC (n=30) | HCs (n=30) | *t*-value | *P*-value |
| --- | --- | --- | --- | --- |
| Mean FA | 0.61 ± 0.01 | 0.61 ± 0.01 | 0.92 | 0.36 |
| Mean MD (×10^-3^), Mean±SD | 0.48 ± 0.01 | 0.47 ± 0.01 | -3.63 | 0.001** |
| Mean AD (×10^-3^), Mean±SD | 0.87 ± 0.02 | 0.85 ± 0.02 | -4.36 | ＜0.001*** |
| Mean RD (×10^-3^), Mean±SD | 0.29 ± 0.01 | 0.28 ± 0.01 | -2.28 | 0.026* |
| Mean NDI, Mean±SD | 0.60 ± 0.02 | 0.63 ± 0.03 | 4.73 | ＜0.001*** |
| Mean ODI, Mean±SD | 0.197 ± 0.005 | 0.204 ± 0.009 | 4.07 | ＜0.001*** |
| Mean ISOVF, Mean±SD | 0.109 ± 0.007 | 0.11 ± 0.008 | 0.36 | 0.72 |

* indicates *P* value < 0.05, ** indicates *P* value < 0.01, *** indicates *P* value *P* < 0.001.

**Supplementary Table S10:** Diffusivities measures and ALPS index of SRC group and HCs group.

|  | SRC (n=30) | HCs (n=30) | *P*-value | *P_FDR_-adjusted* |
| --- | --- | --- | --- | --- |
| Left ALPS index (mm^2^/s) | 1.58 ± 0.13 | 1.48 ± 0.12 | 0.003** | 0.009** |
| Left Dxproj (×10^-4^ mm^2^/s) | 4.60 ± 0.30 | 4.51 ± 0.20 | 0.19 | 0.29 |
| Left Dxassoc (×10^-4^ mm^2^/s) | 4.46 ± 0.41 | 4.16 ± 0.32 | 0.002** | 0.009** |
| Left Dyproj (×10^-4^ mm^2^/s) | 2.93 ± 0.34 | 3.04 ± 0.20 | 0.14 | 0.29 |
| Left Dzassoc (×10^-4^ mm^2^/s) | 2.83 ± 0.22 | 2.84 ± 0.23 | 0.78 | 0.78 |
| Right ALPS index (mm^2^/s) | 1.61 ± 0.14 | 1.51±0.15 | 0.009** | 0.02* |
| Right Dxproj (×10^-4^ mm^2^/s) | 4.58 ± 0.29 | 4.49 ± 0.21 | 0.19 | 0.29 |
| Right Dxassoc (×10^-4^ mm^2^/s) | 4.87 ± 0.40 | 4.53 ± 0.44 | 0.003** | 0.009** |
| Right Dyproj (×10^-4^ mm^2^/s) | 3.10 ± 0.36 | 3.17 ± 0.21 | 0.33 | 0.42 |
| Right Dzassoc (×10^-4^ mm^2^/s) | 2.79 ± 0.25 | 2.82 ± 0.23 | 0.55 | 0.63 |
| Mean index (mm^2^/s) | 1.60 ± 0.13 | 1.50 ± 0.13 | 0.003** | 0.009** |

Data are presented as mean ± SD. SRC = sports-related concussion; HCs = healthy controls; ALPS = along the perivascular space; Dxproj = diffusivities along the x-axis of ROIs within projection fibers; Dxassoc = diffusivities along the x-axis of ROIs within association fibers; Dyproj = diffusivities along the y-axis of ROIs within projection fibers; Dzassoc = diffusivities along the z-axis of ROIs within association fibers.

* Indicates *P* value < 0.05

** indicates *P* value < 0.01.

**Supplementary Table S11:** **Generalized linear mixed models between Clinical assessments and MRI measurements.**

| Clinical assessments | Association *P* values | | | | | | |
| --- | --- | --- | --- | --- | --- | --- | --- |
|  | ALPS-L | ALPS-R | MD | AD | RD | NDI | ODI |
| Duration in sports | 0.88 | 0.80 | 0.39 | 0.13 | 0.70 | 0.35 | 0.11 |
| Number of knockouts | 0.16 | 0.07 | **0.03(+)** | 0.06 | **0.04(+)** | **0.03(-)** | 0.89 |
| HAMA | 0.41 | 0.45 | 0.42 | 0.50 | 0.63 | 0.30 | 0.32 |
| HAMD | 0.23 | 0.40 | 0.65 | 0.99 | 0.96 | 0.38 | 0.11 |
| RAVLT (item 1-5) | **0.007(-)** | 0.30 | 0.15 | 0.35 | 0.12 | 0.16 | 0.49 |
| Forward Digit-span Test | 0.06 | 0.35 | 0.43 | 0.07 | 0.89 | 0.21 | **0.015(+)** |
| Backward Digit-span Test | 0.97 | 0.84 | 0.67 | 0.74 | 0.67 | 0.60 | 0.93 |
| Trail Making Test A | 0.80 | 0.72 | 0.75 | 0.75 | 0.77 | 0.88 | 0.45 |
| Trail Making Test B | 0.20 | 0.25 | 0.92 | 0.94 | 0.92 | 0.75 | 0.94 |

HAMA = Hamilton Anxiety Scale; HAMD = Hamilton Depression Scale; RAVLT = Rey Auditory Verbal Learning Test; + indicates positive association; - indicates negative association.
